# HAZARDOUS WASTE: Pond Algae Sequester Strontium-90

**DOI:** 10.1289/ehp.119-a244

**Published:** 2011-06

**Authors:** Carol Potera

**Affiliations:** **Carol Potera**, based in Montana, has written for *EHP* since 1996. She also writes for *Microbe*, *Genetic Engineering News*, and the *American Journal of Nursing*

Strontium-90 is a radioactive by-product of fission reactions within nuclear reactors that generate electricity. About 3% of the mass of spent nuclear fuel consists of fission products including strontium-90.^1^ Because of its high decay energy and its long half-life of 30 years—it takes hundreds of years to decay naturally to harmless levels—strontium-90 is classified a high-level waste. Strontium-90 deposits in bone and bone marrow, and exposure from contaminated food and water is linked to bone cancer and leukemia.^2^ Now Derk Joester, an assistant professor of materials science and engineering at Northwestern University in Evanston, Illinois, and his colleagues have found that common freshwater green algae sequester strontium into insoluble crystals, offering a possible way to separate strontium-90 from less hazardous components of nuclear waste.^3^

*Closterium moniliferum*, a ubiquitous bright green pond alga, forms crystals composed of strontium, barium, and sulfate. The crescent-shaped algae store the crystals in tiny vacuoles. Barium is necessary for the organism to deposit strontium, and the Northwestern team found that varying the ratio of barium to strontium in water boosted the amount of strontium captured in crystals by a factor of up to 150.^3^ This enhanced the strontium selectivity of the process.

Nonradioactive strontium was used for the proof-of-concept laboratory experiments. Whether *C. moniliferum* tolerates radioactive strontium-90 needs to be determined, but the authors point out these organisms “have proven to be resistant to harsh environments such as extreme temperature, acidic pH, low nutrient availability, and light limitation.”^3^

*C. moniliferum* also prefers strontium to calcium. This is important because calcium, a harmless mineral, is found in nuclear waste along with strontium. Plants tested for bioremediation do not differentiate between strontium and calcium, so they become saturated with the latter simply because it is more abundant.^4^ But *C. moniliferum* does differentiate: “Algae avoid this problem by actively excreting calcium during crystal formation,” Joester says. He adds that algae potentially could become direct bioremediation agents, and understanding how they lock up strontium could lead to better engineered microbes.

The Northwestern team envisions a filtration system where algae would precipitate crystals in hours or days. The crystals would be harvested, then incinerated to remove organic matter. The remaining concentrated crystals would be fused into glass blocks (“vitrified”) for safe storage, according to first author Minna Krejci, a Ph.D. candidate at Northwestern University.

Some nuclear waste is already vitrified,^1^ but Joester says the sheer volume of nuclear waste makes it economically unfeasible to contain everything in glass blocks. The U.S. Department of Energy estimates the cost to process all radioactive waste currently stored in the United States at $50 billion.^5^

“These results look very promising for the use of green algae for bioremediation,” says Belinda Sturm, an environmental engineer at the University of Kansas, Lawrence. However, algae grown for biofuels are expensive to harvest at large scale, and similar challenges may apply to algae that clean up nuclear waste. “This does not negate their potential but emphasizes the need for further study,” Sturm says.

If sequestration proves successful, algae may help to recover strontium-90 dispersed in oceans, lakes, or rivers after nuclear accidents, such as leaks from the Fukushima nuclear power plant in Japan.^6^ Perhaps algae could be designed to sink to the bottom, allowing strontium-90 to decay without entering the food chain, or floating algae could be skimmed off the surface and contained, suggests Roger Blomquist, principal nuclear engineer at Argonne National Laboratory.

## Figures and Tables

**Figure:**
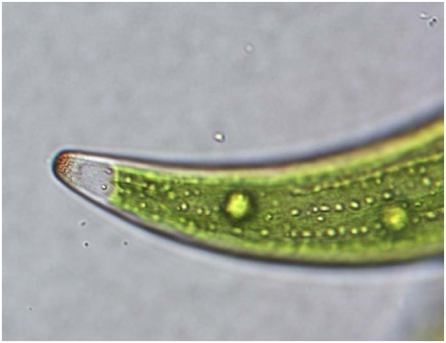
*C. moniliferium* cell has deposited strontium-bearing barite crystals in the gray vacuole at the alga’s tip.
